# Reduced Levels of ATP Synthase Subunit ATP5F1A Correlate with Earlier-Onset Prostate Cancer

**DOI:** 10.1155/2018/1347174

**Published:** 2018-11-14

**Authors:** René G. Feichtinger, Georg Schäfer, Christof Seifarth, Johannes A. Mayr, Barbara Kofler, Helmut Klocker

**Affiliations:** ^1^Research Program for Receptor Biochemistry and Tumor Metabolism, Department of Pediatrics, University Hospital Salzburg, Paracelsus Medical University, 5020 Salzburg, Austria; ^2^Department of Pediatrics, University Hospital Salzburg, Paracelsus Medical University, 5020 Salzburg, Austria; ^3^Division of Experimental Urology, Department of Urology, Medical University of Innsbruck, 6020 Innsbruck, Austria; ^4^Department of Pathology, Medical University of Innsbruck, 6020 Innsbruck, Austria

## Abstract

Switching of cellular energy production from oxidative phosphorylation (OXPHOS) to aerobic glycolysis occurs in many types of tumors. However, the significance of energy metabolism for the development of prostate carcinoma is poorly understood. We investigated the expression of OXPHOS complexes in 94 human prostate carcinomas and paired benign tissue using immunohistochemistry. Overall mitochondrial mass was upregulated in carcinomas compared to benign prostate tissue in all Gleason grades. A significant direct correlation between the expression of OXPHOS complexes I, II, and V and the Gleason score was observed. However, 17% of prostate carcinomas and 18% of benign prostate tissues showed isolated or combined deficiency of OXPHOS complexes (one deficiency in 12% of the tumors, combined deficiencies in 5%). Complex I was absent in 9% of the samples, with only parts of the tumor affected. ATP5F1A, a complex V protein, was the most frequently affected subunit, in 10% of tumors and 11% of benign prostate tissues (but not both tissues in any single patient). A possible role of complex V in prostate cancer development is suggested by the significant positive correlation of ATP5F1A levels with earlier-onset prostate cancer (age at diagnosis and at prostatectomy) and free PSA percentage. The relatively high percentage (17%) of prostate carcinomas with regional foci of partial OXPHOS complex deficiencies could have important therapeutic implications.

## 1. Introduction

Metabolism, especially energy metabolism, is a hot topic in tumor biology. Many tumor entities are characterized by deficiencies and reprogramming of mitochondrial energy metabolism. Sometimes, a single tumor entity can be divided into subgroups, where one group shows high levels of oxidative phosphorylation (OXPHOS) and the other is deficient in one or more OXPHOS complexes, as found in melanomas [1]. Some tumor entities are very homogeneous in their OXPHOS signature; for example, neuroblastomas and renal cell carcinomas both show significant reductions of all OXPHOS complexes. In contrast, many other carcinomas (e.g., gastric carcinomas and colorectal carcinomas) show a relatively constant number of OXPHOS defects [1–13]. Although the mode of downregulation of OXPHOS can vary (loss of mitochondria, loss of all complexes, isolated and combined deficiencies), nearly all tumors have one feature in common—disruption of respiratory chain complex I [1–13].

Mitochondrial complex I is clearly at the center of the OXPHOS deficiency landscape in cancer, and there are several explanations for this. Subunits of complex I, namely, NDUFS3 and NDUFS1, can be cleaved by granzyme A or granzyme B and caspase 3 to induce apoptosis [14, 15]. A lack of complex I might help shield tumor cells from apoptosis. Secondly, complex I is the most elaborate mitochondrial multisubunit protein, potentially making it more prone to damage [16]. Statistically, the large number of subunits might also explain the higher incidence of genetic hits affecting complex I. It is also reported that complex I is the main production site of reactive oxygen species (ROS) within the respiratory chain [17]. Since ROS immediately react with biomolecules, complex I might be the first target of its own generated ROS. ROS also attack mtDNA, including the 7 mitochondrially encoded complex I subunits [18].

Combined OXPHOS deficiencies are the most frequent alteration with regard to tumors and patients with mitochondrial disease [19]. Fifty-seven percent of all patients diagnosed in our center with a mitochondrial disease show a combined reduction of OXPHOS complexes [19]. Combined OXPHOS deficiencies can arise from mitochondrial tRNA mutations, mtDNA deletions, and mtDNA depletion [19]. Since respiratory chain complexes are further organized into super/megacomplexes, mutations in complex III subunits typically also lead to secondary loss of complex I [20]. Furthermore, mutations in a number of genes involved in mitochondrial biogenesis, architecture, or protein transport can cause combined defects [19]. In total, mutations in more than 250 genes cause mitochondrial disorders [19]. Interestingly, unlike mutations in respiratory chain subunits, mutations affecting other mitochondrial proteins are generally not associated with tumors (exceptions include IDH2/isocitrate dehydrogenase 2, FH/fumarate dehydrogenase, and DGUOK/deoxyguanosine kinase) [21–23].

Prostate cancer is the most frequently diagnosed cancer in males in developed countries and responsible for more than 300,000 annual cancer deaths worldwide [24]. Mitochondrial DNA alterations are frequent in prostate cancer and have been correlated to pathological features, tumor progression, and worse outcomes [25–27]. The aim of the present study was to characterize the metabolic phenotype of prostate carcinomas and corresponding benign prostate tissue by immunohistochemical (IHC) staining of mitochondria and individual mitochondrial complexes. Previously, we demonstrated that semiquantitative IHC of homogeneous tissue samples correlates well with functional analysis, as the OXPHOS system is mainly regulated via protein amount [2, 3, 5–8, 13, 28]. Therefore, IHC of heterogeneous samples is the method of choice as it accurately reflects the *in vivo* situation at the cellular level and can detect small areas of OXPHOS deficiency in tumor samples.

## 2. Material and Methods

### 2.1. Ethics

Human tumors were obtained from the Institute of Pathology, Medical University Innsbruck. The study was performed according to the Austrian Gene Technology Act. Experiments were conducted in accordance with the Helsinki Declaration of 1975 (revised 2013) and the guidelines of the local ethics committee, being no clinical drug trial or epidemiological investigation. All patients signed an informed consent document concerning the surgical intervention. Furthermore, the study did not extend to the examination of individual case records. Patient anonymity was ensured at all times. The use of the archived tissue samples was approved by the ethics committee of the Medical University Innsbruck (AN 3174, AN 4837).

### 2.2. Samples

To evaluate differences in expression between malignant and benign prostate tissues, we constructed a tissue microarray (TMA) of formalin-fixed, paraffin-embedded tissue blocks from 94 previously untreated prostate cancer patients who had undergone radical prostatectomy after tumor diagnosis in a PSA screening program performed in Tyrol by the Department of Urology, Medical University Innsbruck [29]. The TMA was assembled using a manual tissue arrayer (Beecher Instruments, Sun Prairie, WI). Four punches of each case, 3 from the tumor and 1 from a benign area, were evaluated. In many cases, tissue cores contained tumor as well as benign regions.

### 2.3. AMACR/p63 Staining

Hematoxylin and eosin staining, basal cell maker p63 and tumor cell marker alpha-methylacyl-CoA racemase (AMACR) immunohistochemistry (IHC) double stains were used to confirm the histological diagnosis. IHC was performed on a Discovery-XT-automated staining device (Ventana, Tucson, AZ) using instrument standard protocols. Antibodies, suppliers, catalog numbers, and concentrations used were as follows: anti-p63, Sigma-Aldrich, #P3362, 1 : 200; anti-AMACR, Dako (Vienna, Austria), #M3616, 1 : 200. DAB (3,3′-diaminobenzidine; brown) was used for visualization of AMACR and nitroblue tetrazolium chloride (blue) for staining of p63.

### 2.4. Immunohistochemical Staining of OXPHOS Complex Subunits and Porin of FFPE Tissues

For IHC, the following antibodies were used: complex I subunit NDUFS4 (mouse monoclonal, 1 : 1000; Abcam, Cambridge, UK), complex II subunit SDHA (mouse monoclonal, 1 : 2000; MitoSciences, Eugene, Oregon), complex III subunit UQCRC2 (mouse monoclonal, 1 : 1500; MitoSciences), complex IV subunit MT-COI (mouse monoclonal, 1 : 1000; MitoSciences), complex V subunit ATP5F1A (mouse monoclonal, 1 : 2000; MitoSciences), and VDAC1 (mouse monoclonal, 1 : 3000; MitoSciences). All antibodies were diluted in Dako antibody diluent with background-reducing components (Dako, Glostrup, Denmark). IHC was performed as described previously [13]. For antigen retrieval, the sections were immersed for 45 min in 1 mM EDTA, 0.05% Tween-20, pH 8, at 95°C. Tissue sections were incubated for 30 min with the above-mentioned primary antibodies. The staining intensities of the tumor and control tissues were determined by two examiners using a stereomicroscope. Staining intensities were rated using a scoring system ranging from 0 to 4, with 0 indicating no staining, 1 mild, 2 moderate, 3 strong, and 4 very strong staining. Four punches of each tissue sample were analyzed, and for each punch, the lowest and highest intensities were scored. Staining intensities of each tissue punch, mean staining intensities of each sample, and clinical data are given in Supplementary Tables 1A and 1B. For statistical analysis (*t*-test, ANOVA, and correlations), the mean of the staining intensities of the four punches was used. For the frequency distribution, the lowest staining intensity of each sample was analyzed. The specificity of the antibodies used was previously shown in numerous articles by Western blot analysis: ([6]; Figure 3), ([5], Figure 2); NDUFS4 ([6]; Figure 3), ([5], Figure 2); SDHA ([6]; Figure 3), ([5], Figure 2); UQCRC2 ([6]; Figure 3), ([5], Figure 2); MT-CO1 ([30], Figure 2); ATP5F1A ([6]; Figure 3), ([31]; Figure 3). The punches were also stained with AMACR/p63 to distinguish between tumor and benign prostate tissue. All antibodies are suitable for the detection of assembly defects. If these subunits are missing, the respective complexes are not assembled and, therefore, no function is present. The antibodies were used to detect numerous defects caused by pathogenic mutations. The activity of the OXPHOS enzymes was determined in previous studies, underlining that the amount of protein and the level of activity are highly correlated and showing that loss of the subunits causes loss of activity [2, 3, 5, 7, 20, 28, 31, 32].

### 2.5. Statistical Analysis

For the comparison of tumors and benign prostate tissue, a *t*-test was applied. For multiple comparisons of tumors with different Gleason scores, one-way ANOVA and Bonferroni's correction were applied. Pearson's correlation was applied to analyze potential associations between the Gleason score and OXPHOS complex expression. In addition, the frequency distribution of the staining intensities was calculated.

## 3. Results

### 3.1. Increased Mitochondrial Biogenesis and Consistent Upregulation of OXPHOS Complexes in Carcinomas Compared to Adjacent Benign Prostate Tissue

**Figure 1:**
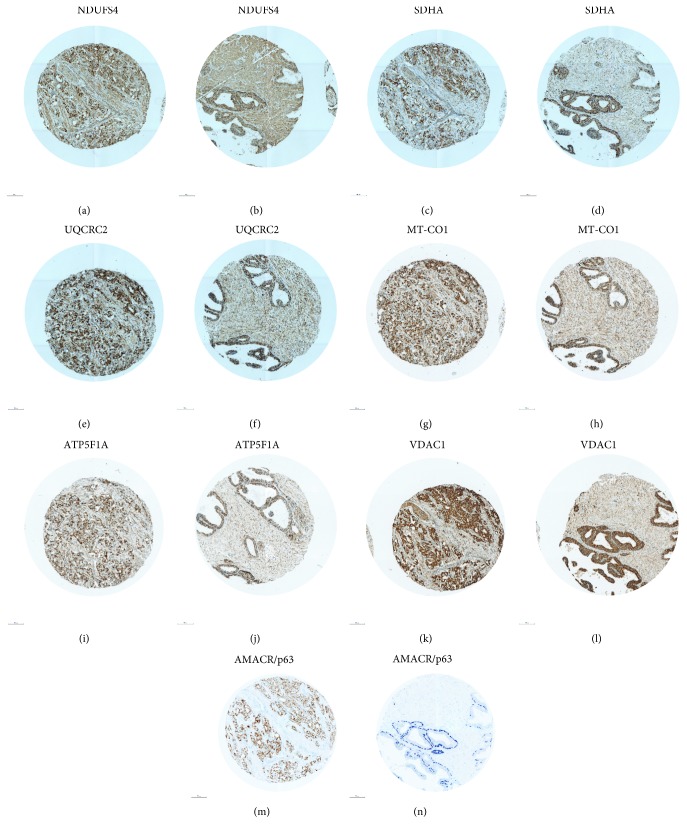
Staining of OXPHOS complexes, VDAC1, and AMACR/p63 in a prostate carcinoma of Gleason score 9 and adjacent benign prostate tissue. (a, b) NDUFS4; (c, d) SDHA; (e, f) UQCRC2; (g, h) MT-CO1; (i, j) ATP5F1A; (k, l) VDAC1; and (m, n) AMACR/p63. (a, c, e, g, i, k, m) carcinoma and (b, d, f, h, j, l, n) hyperplasia. The punches are 0.6 mm in diameter. AMACR/p63 staining was used to visualize carcinoma cells (brown) and benign prostate tissue (blue).

**Figure 2:**
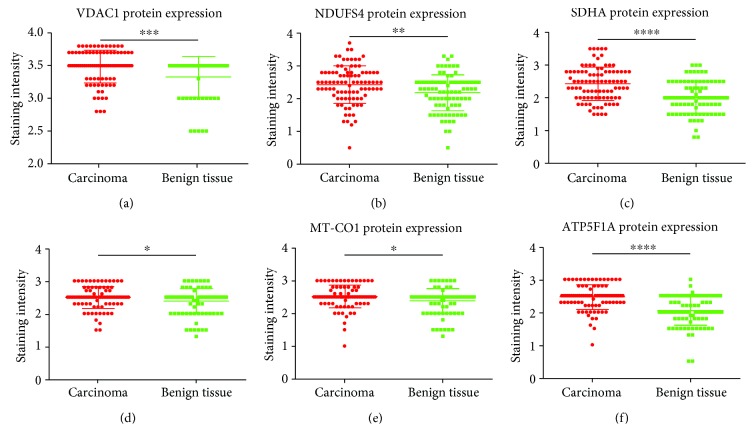
OXPHOS complex expression in prostate carcinomas and corresponding benign prostate tissue. (a) VDAC1, (b) NDUFS4, (c) SDHA, (d) UQCRC2, (e) MT-CO1, and (f) ATP5F1A. The intensities of the stainings are given as the median ± SD. ^∗∗∗∗^ *p* < 0.0001, ^∗∗∗^ *p* < 0.001, ^∗∗^ *p* < 0.01, and ^∗^ *p* < 0.05.

To elucidate if prostate carcinomas and adjacent benign prostate tissue differ in terms of protein expression of the OXPHOS complexes and mitochondrial biogenesis, we immunohistochemically stained prostate carcinomas (*n*~94) and corresponding benign prostate tissue (*n*~89). The benign and cancerous tissues were distinguished by AMCR/p63 staining: the carcinoma stained brown and the epithelium of benign glands stained dark blue (Figure 1). We also stained for VDAC1, a protein of the outer mitochondrial membrane, as an indicator of mitochondrial mass and therefore mitochondrial biogenesis. Overall, we detected a significantly higher expression level of VDAC1 (*p* = 0.0009) in carcinomas compared to adjacent benign prostate tissue (*n* = 62) (Figure 2(a)), although the latter showed a significantly higher proportion of samples with “very strong staining” (score = 4) (24% of benign tissue vs. 6% of tumor tissue). In accordance, the staining levels of NDUFS4 (*p* = 0.01), SDHA (*p* < 0.0001), UQCRC2 (*p* < 0.05), MT-CO1 (*p* < 0.05), and ATP5F1A (*p* < 0.0001) were all significantly higher in the prostate carcinomas compared to their benign counterparts (Figures 2(b)–2(f)). Since altogether the OXPHOS complexes and VDAC1 are increased in carcinomas, we conclude that mitochondrial mass is higher in carcinomas than in benign prostate tissue.

### 3.2. Comparison of Prostate Carcinomas and Adjacent Benign Prostate Tissue with respect to Tumor Malignancy

To test if the altered expression of subunits of the OXPHOS complexes is associated with malignancy of prostate carcinomas, we compared IHC immunoreactivities among the different pathological Gleason score (GS) grades. We found no clear-cut association of complex marker expression with GS grade, except in the case of GS 7 tumors, which showed increased expression of all mitochondrial complex markers. Nonetheless, in agreement with the generalized increase in mitochondrial mass observed in prostate carcinomas, we detected overall trends to higher expression in all GS grades (Supplementary Table 2). The mean staining intensities over all tissue punches from each tumor case revealed no differences between prostate carcinomas of different GS grades for VDAC1, SDHA, MT-CO1, and ATP5F1A (Supplementary Figure 1). For NDUFS4, both GS 7 and 9 carcinomas showed significantly higher expression than GS 6 and 8 tumors (Supplementary Figure 1). A correlation analysis revealed increased expression with Gleason scores for NDUFS4 (*p* < 0.05; *R* = 0.22), SDHA (*p* < 0.05; *R* = 0.25), and ATP5F1A (*p* < 0.05; *R* = 0.22).

### 3.3. Frequency of OXPHOS Deficiencies

The overall analysis of staining intensities often masks important results when only the averages are used for calculation and variations within the analyzed tissue specimen are ignored. Tumors, especially prostate tumors, are heterogeneous, and alterations of protein expression in small areas of the tissue, indicating regional loss of expression, need to be considered separately. We analyzed expression heterogeneity in more detail in the four cores of each case to uncover regional OXPHOS deficiencies. The lowest staining intensity for each tumor or benign prostate tissue was identified, and the frequency distribution was evaluated to elucidate the percentage of specimens exhibiting OXPHOS deficiency (staining intensity = 0).

**Figure 3:**
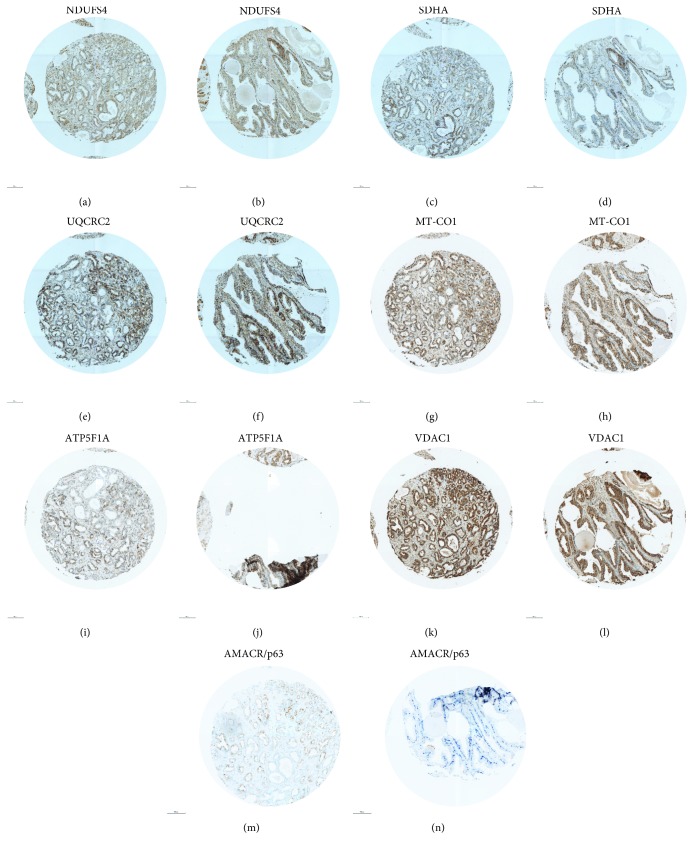
Staining of OXPHOS complexes, VDAC1, and AMACR/p63 in a prostate carcinoma with partial loss of ATP5F1A and adjacent benign prostate tissue. (a, b) NDUFS4; (c, d) SDHA; (e, f) UQCRC2; (g, h) MT-CO1; (i, j) ATP5F1A; (k, l) VDAC1; and (m, n) AMACR/p63. (a, c, e, g, i, k, m) carcinoma and (b, d, f, h, j, l, n) benign prostate tissue. The punches are 0.6 mm in diameter. AMACR/p63 staining was used to visualize carcinoma cells (brown) and benign prostate tissue (blue).

**Figure 4:**
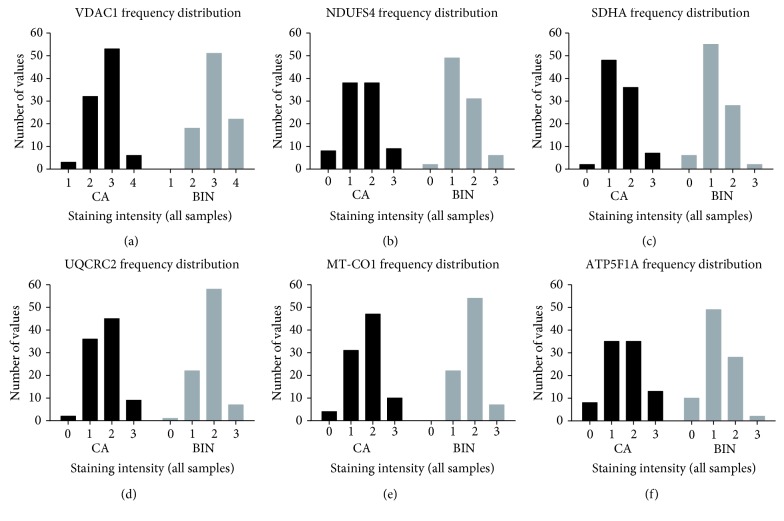
Frequency distribution of the lowest staining intensities found in prostate carcinoma and corresponding benign prostate tissue punches. (a) VDAC1, (b) NDUFS4, (c) SDHA, (d) UQCRC2, (e) MT-CO1, and (f) ATP5F1A. The frequencies are given in percent. The staining intensities are as follows: 0 = no staining, 1 = weak staining, 2 = moderate staining, 3 = strong staining, and 4 = very strong staining. CA, prostate carcinoma; BIN, benign prostate tissue.

The tumors of 16 patients (17%) showed deficiencies of respiratory chain complexes. Eleven tumors showed deficiencies in one complex only, three tumors exhibited deficiencies in two complexes, and two tumors exhibited losses of three and four complexes, respectively. The frequency of partial mitochondrial complex loss in benign tissue was 18%, similar to that in tumors. Deficiency of complex I was present in 9% of carcinomas compared to 2% of benign prostate tissue samples (Figure 3, Supplementary Figures 2 and 3). Deficiency of SDHA (2% vs. 7%) and ATP5F1A (10% vs. 11%) (Figure 3) was more frequent in benign prostate tissue (Figure 4). ATP5F1A deficiency was never observed in both carcinoma and adjacent benign prostate tissue. UQCRC2 was lost in 2% of the tumors and in 1% of the benign prostate tissue, whereas MT-CO1 loss was observed only in carcinomas (4%). There was no clear association of complex loss with the tumor Gleason score (Supplementary Figure 2).

### 3.4. Correlation of Clinical Parameters and the Expression of OXPHOS Complexes

**Figure 5:**
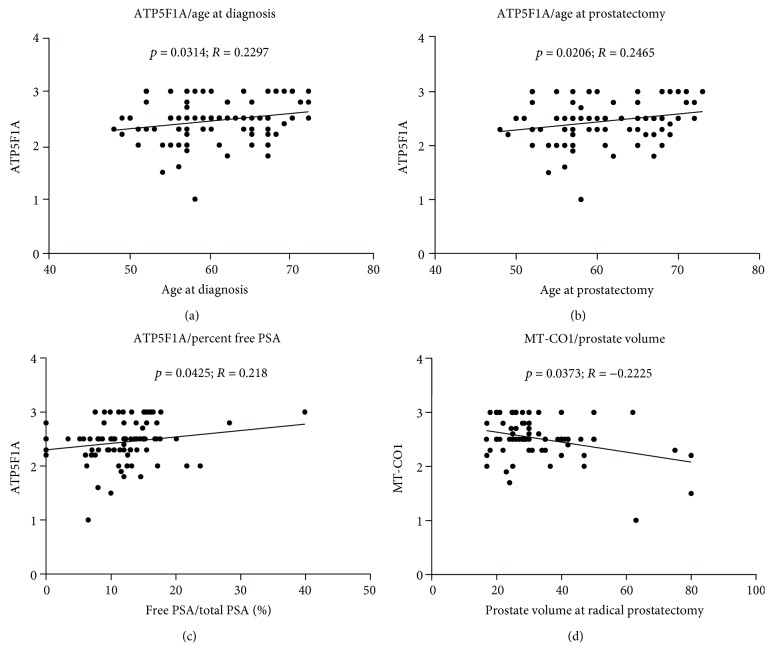
Correlation between clinical parameters and the levels of OXPHOS complexes. Correlation between ATP5F1A level and age at diagnosis (a), age at prostatectomy (b), and percent free PSA in relationship to total PSA (c). (d) Correlation between MT-CO1 levels and prostate volume at prostatectomy.

We analyzed the correlation of the expression of OXPHOS complexes and porin with age at prostatectomy, pathological tumor stage, serum PSA level, free to total PSA ratio, prostate size at radical prostatectomy, and time until biochemical tumor recurrence (PSA progression). A significant positive correlation was present between complex V expression and patient age at diagnosis (*p* = 0.0314; *R* = 0.2297) and at prostatectomy (*p* = 0.0206; *R* = 0.2465) (Figures 5(a) and 5(b)). Furthermore, complex V expression was significantly higher in individuals with high free to total PSA ratios (*p* = 0.0425; *R* = 0.218) (Figure 5(c)), whereas a negative correlation between prostate volume at prostatectomy and complex IV was found (*p* = 0.0373; *R* = −0.225) (Figure 5(d)).

No differences of OXPHOS complex levels in tumors were observed with respect to pathological stage, TNM (T: size or direct extent of the primary tumor; N: degree of spread to regional lymph nodes; and M: presence of distant metastasis) or biochemical tumor recurrence.

## 4. Discussion

Analysis of mitochondrial mass and individual mitochondrial OXPHOS complexes using surrogate protein markers detected by IHC revealed an increased mitochondrial mass in prostate carcinomas in comparison to their benign tissue counterparts. VDAC1 was used as a marker for the mitochondrial mass. It was previously shown that it correlates excellently with citrate synthase activity, which is also used as a marker for the mitochondrial amount in functional studies [2, 3, 5, 7, 20, 28]. mtDNA is not a reliable marker for mitochondrial mass since many solid tumor entities have a high or normal mitochondrial mass but a reduced mtDNA copy number [5, 7]. Also, patients with mitochondrial diseases affecting muscle argue against this (TK2, SUCLA2, SUCLG1, RRM2B, DGUOK, and TYMP) [32, 33]. Patients with an mtDNA depletion disorder show a normal mitochondrial amount but a reduction of one or more OXPHOS complexes (e.g., DGUOK) [32, 33].

In addition, our analysis identified a subgroup of about one-sixth of the analyzed prostate carcinoma specimens harboring areas of isolated or combined loss of OXPHOS complexes.

The most frequent alterations of the OXPHOS system in our cohort were the loss of complex I and complex V. The absence of complex I protein and potentially pathogenic mutations of mitochondrial complex I genes have been consistently reported for numerous tumor entities [1, 4–6, 10, 12, 13, 28, 33]. A very recent study sequenced mtDNA in 384 prostate carcinoma patients and identified 129 nonsynonymous mitochondrial single-nucleotide variants in protein-coding regions, including six premature stop codons and two mutated stop codons [25]. The most frequently affected protein-coding gene was MT-ND5 (30 out of 157 mitochondrial synonymous and nonsynonymous single nucleotide variants). In addition, 8 mutations in the anticodon stem of mitochondrial tRNA genes were present. As a consequence, combined OXPHOS complex deficiencies would be expected, as observed in 5 cases in our study. According to a recent review, 749 mtDNA mutations have been described for prostate cancer [34]. Only 80 of these were found in two or more patients, 15 of which are potentially pathogenic according to commonly used prediction tools [34]. We are not aware of any previous study correlating nuclear OXPHOS subunit gene alterations to the frequency of OXPHOS deficiency in prostate carcinomas.

In line with many other studies, the loss of complex I was the most frequent event in our cohort of primary prostate tumors. An explanation might be that complex I deficiency confers a selective advantage on tumor cells since it is part of an important apoptosis pathway. The NDUFS1 subunit of complex I can be cleaved by caspase 3 to induce apoptosis [15].

Interestingly, ATP synthase (complex V) staining was decreased in a substantial number of prostate carcinomas (10%) and benign prostate tissues (11%). We have previously shown that the loss of complex V is a rare event in different solid tumors [1, 4–6, 10, 12, 13, 28, 33]. In contrast to these tumor entities, a significant percentage of prostate tumors is characterized by this relatively rare bioenergetic phenotype. Overall, the level of complex V subunit ATP5F1A was increased in our prostate cancer cohort. Up- or downregulation of specific subunits of ATP synthase was reported depending on the tumor entity [35–40]. Increased levels of ATP synthase subunits *α* and d were present in 76–79% of colorectal carcinomas [41]. ATP synthase d was significantly overexpressed in 93 lung adenocarcinomas [39]. We also found that complex V and complex II usually correlate with mitochondrial mass, as indicated by VDAC1 and citrate synthase activity [4, 5, 12, 13, 28, 42].

To our knowledge, this is the first report of the total lack of complex V in a subset of prostate tumors. Interestingly, complex V defects are very rare among patients with mitochondrial diseases compared to defects of the other four complexes of the OXPHOS system. Mutations in patients have been reported only for the mitochondrial genes MT-ATP6 and MT-ATP8 and the nuclear genes ATP5F1A and ATP5E [43–45]. In this context, the ATP synthase inhibitory factor 1 (IF_1_) is of interest because it is upregulated in many human carcinomas and implicated in the control of mitochondrial bioenergetics and structure by regulating the activity and oligomerization of ATP synthase [46–48]. IF_1_ suppresses programmed cell death, thereby enhancing tumor invasion and chemoresistance. Indeed, a possible role of complex V in prostate cancer development is also suggested by the significant positive correlation we detected between ATP5F1A level and age at diagnosis/prostatectomy as well as ATP5F1A level and percentage of free PSA in relationship to total PSA.

One of every six men will be diagnosed with prostate carcinoma during his lifetime (https://www.cancer.org/cancer/prostate-cancer/about/key-statistics.html), and prostate cancer accounts for 8% of all new cancer cases. Therefore, the finding that a high percentage of prostate carcinomas and the corresponding benign prostate tissues show OXPHOS deficiency warrants consideration in therapeutic strategies.

## 5. Conclusions

Based on our own studies, we estimate that at least 10% of all tumor cases show low levels of OXPHOS similar to those we detected in prostate carcinomas. Since most diagnosed tumors are carcinomas, this is a very conservative estimate. Therefore, the therapeutic implications of OXPHOS deficiencies are potentially enormous because these tumors should be prone to metabolic therapies used to selectively kill tumor cells.

## Data Availability

The data used to support the findings of this study are included in the article.

## Supplementary Materials

Supplementary Materials Supplementary Figure 1: OXPHOS complex expression in prostate carcinomas of different Gleason scores. (a) VDAC1, (b) NDUFS4, (c) SDHA, (d) UQCRC2, (e) MT-CO1, and (f) ATP5F1A. The intensities of the stainings are given as the median ± SD. ^∗∗^ *p* < 0.01 and ^∗^ *p* < 0.05. Supplementary Figure 2: frequency distribution of the lowest staining intensities found in prostate carcinoma tissue punches of different Gleason scores. (a) VDAC1, (b) NDUFS4, (c) SDHA, (d) UQCRC2, (e) MT-CO1, and (f) ATP5F1A. The frequencies are given as percentages. The staining intensities are as follows: 0 = no staining, 1 = weak staining, 2 = moderate staining, 3 = strong staining, and 4 = very strong staining. Supplementary Figure 3: staining of OXPHOS complexes, VDAC1, and AMACR/p63 in a prostate carcinoma of Gleason score 7 and adjacent benign prostate tissue. (a, b) NDUFS4; (c, d) SDHA; (e, f) UQCRC2; (g, h) MT-CO1; (i, j) ATP5F1A; (k, l) VDAC1; and (m, n) AMACR/p63. (a, c, e, g, i, k) carcinoma and (b, d, f, h, j, l, n) benign prostate tissue. The punches are 0.6 mm in diameter. AMACR/p63 staining was used to visualize carcinoma cells (brown) and benign prostate tissue (blue). Supplementary Table 1: staining intensities of each tissue punch and mean staining intensities with clinical data. Supplementary Table 2: comparison of the expression levels of OXPHOS subunits and VDAC1 in benign prostate tissue and carcinomas of different Gleason scores.
