# Supplementary material for: Reduced Levels of ATP Synthase Subunit ATP5F1A Correlate with Earlier-Onset Prostate Cancer
Source: Oxid Med Cell Longev. 2018 Nov 14;2018:1347174. doi: 10.1155/2018/1347174 (PMC6261400; doi:10.1155/2018/1347174)
Supplement: Supplementary Materials — Supplementary Figure 1: OXPHOS complex expression in prostate carcinomas of different Gleason scores. (a) VDAC1, (b) NDUFS4, (c) SDHA, (d) UQCRC2, (e) MT-CO1, and (f) ATP5F1A. The intensities of the stainings are given as the median ± SD. ∗∗ p < 0.01 and ∗ p < 0.05. Supplementary Figure 2: frequency distribution of the lowest staining intensities found in prostate carcinoma tissue punches of different Gleason scores. (a) VDAC1, (b) NDUFS4, (c) SDHA, (d) UQCRC2, (e) MT-CO1, and (f) ATP5F1A. The frequencies are given as percentages. The staining intensities are as follows: 0 = no staining, 1 = weak staining, 2 = moderate staining, 3 = strong staining, and 4 = very strong staining. Supplementary Figure 3: staining of OXPHOS complexes, VDAC1, and AMACR/p63 in a prostate carcinoma of Gleason score 7 and adjacent benign prostate tissue. (a, b) NDUFS4; (c, d) SDHA; (e, f) UQCRC2; (g, h) MT-CO1; (i, j) ATP5F1A; (k, l) VDAC1; and (m, n) AMACR/p63. (a, c, e, g, i, k) carcinoma and (b, d, f, h, j, l, n) benign prostate tissue. The punches are 0.6 mm in diameter. AMACR/p63 staining was used to visualize carcinoma cells (brown) and benign prostate tissue (blue). Supplementary Table 1: staining intensities of each tissue punch and mean staining intensities with clinical data. Supplementary Table 2: comparison of the expression levels of OXPHOS subunits and VDAC1 in benign prostate tissue and carcinomas of different Gleason scores. [file 1347174.f1.pdf]

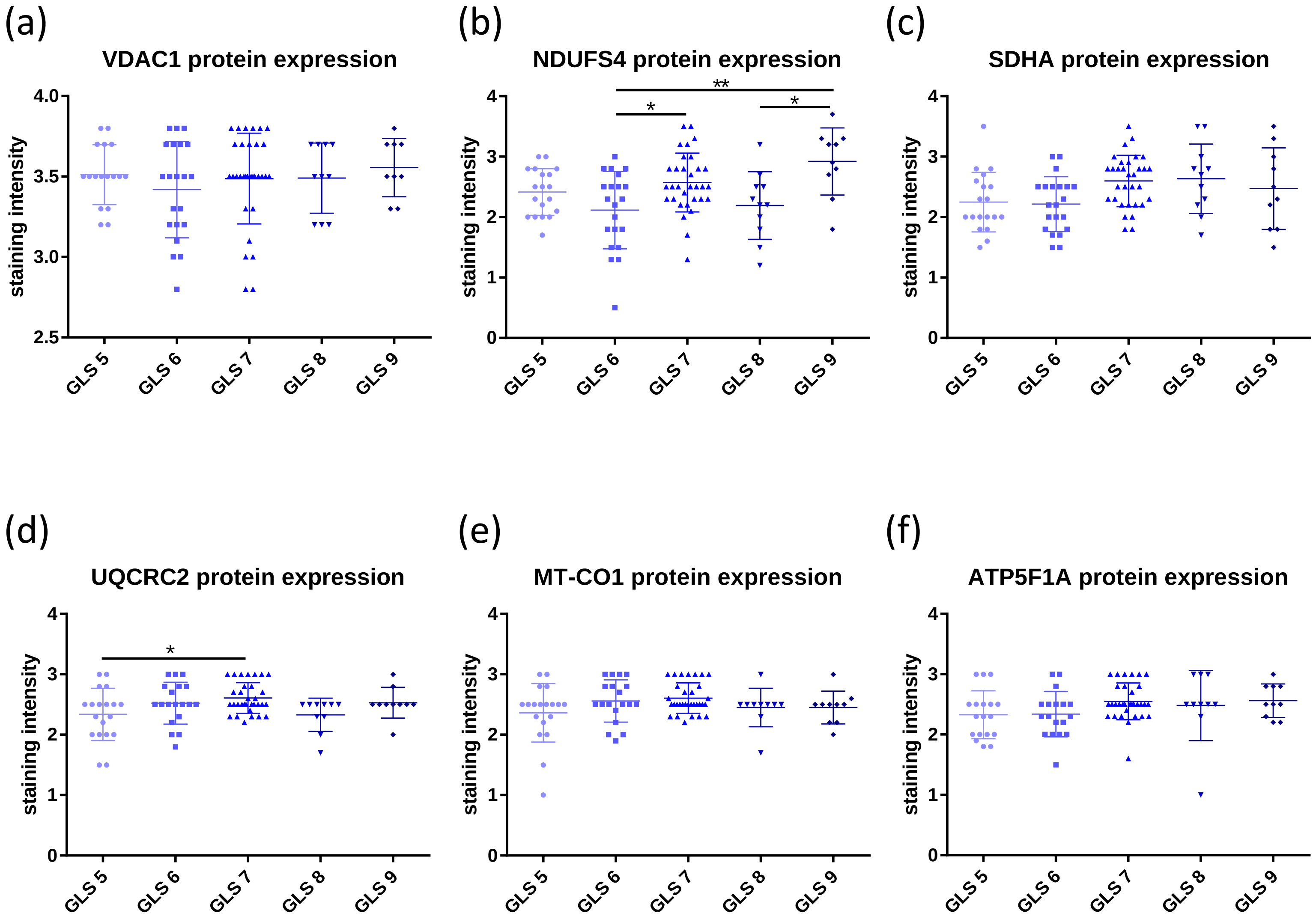

Supplementary figure 1: OXPHOS complex expression in prostate carcinomas of different Gleason scores. (a) VDAC1, (b) NDUFS4, (c) SDHA, (d) UQCRC2, (e) MT-CO1, (f) ATP5F1A. The intensities of the stainings are given as median ± SD. ***p<0.001, **p>0.01, *p>0.05.

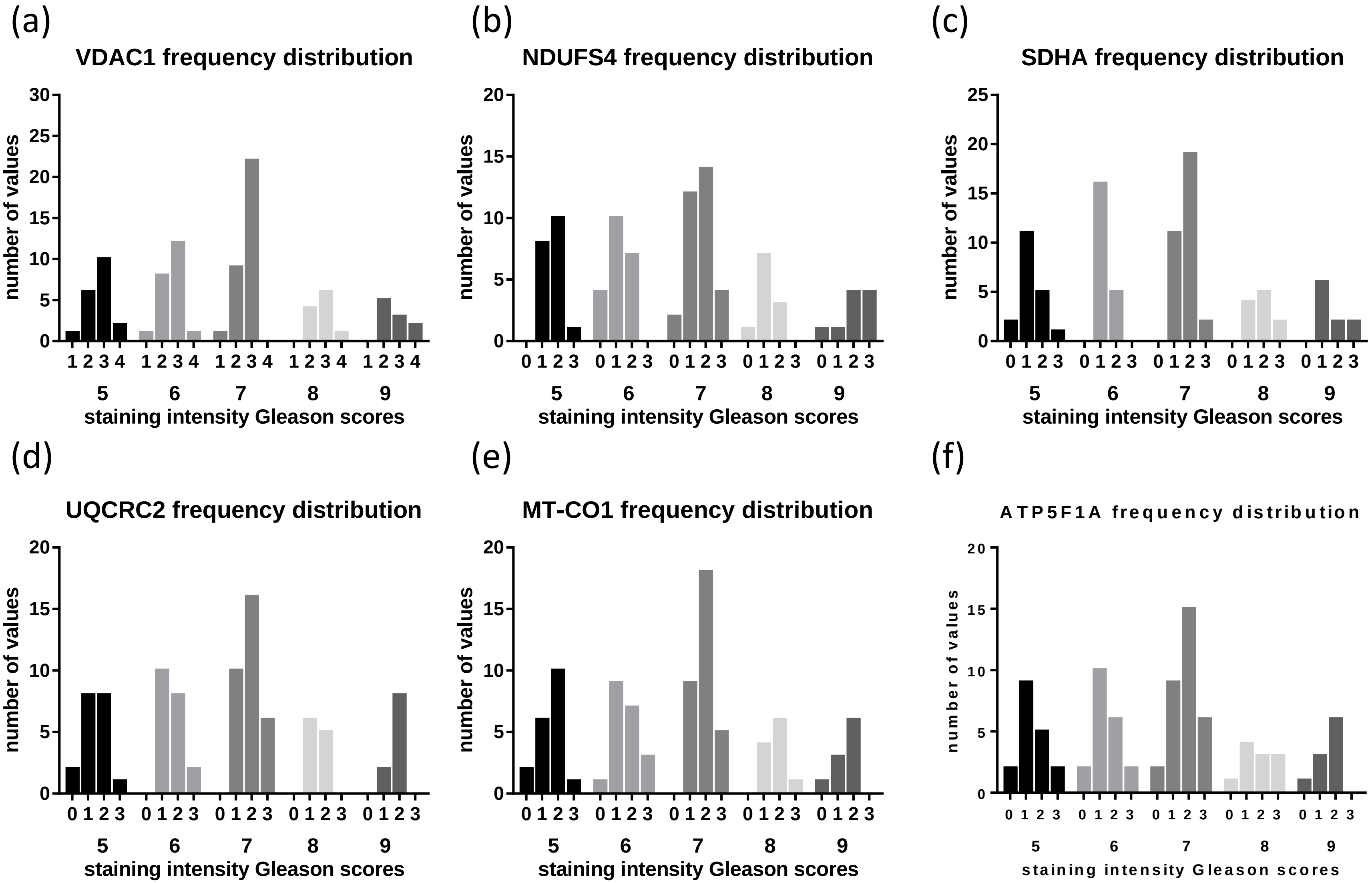

Supplementary figure 2: Frequency distribution of the lowest staining intensities found in prostate carcinoma tissue punches of different Gleason scores. (a) VDAC1, (b) NDUFS4, (c) SDHA, (d) UQCRC2, (e) MT-CO1, (f) ATP5F1A. The frequencies are given in percent. The staining intensities are: 0 = no staining, 1 = weak staining, 2 = moderate staining, 3 = strong staining, 4 = very strong staining.

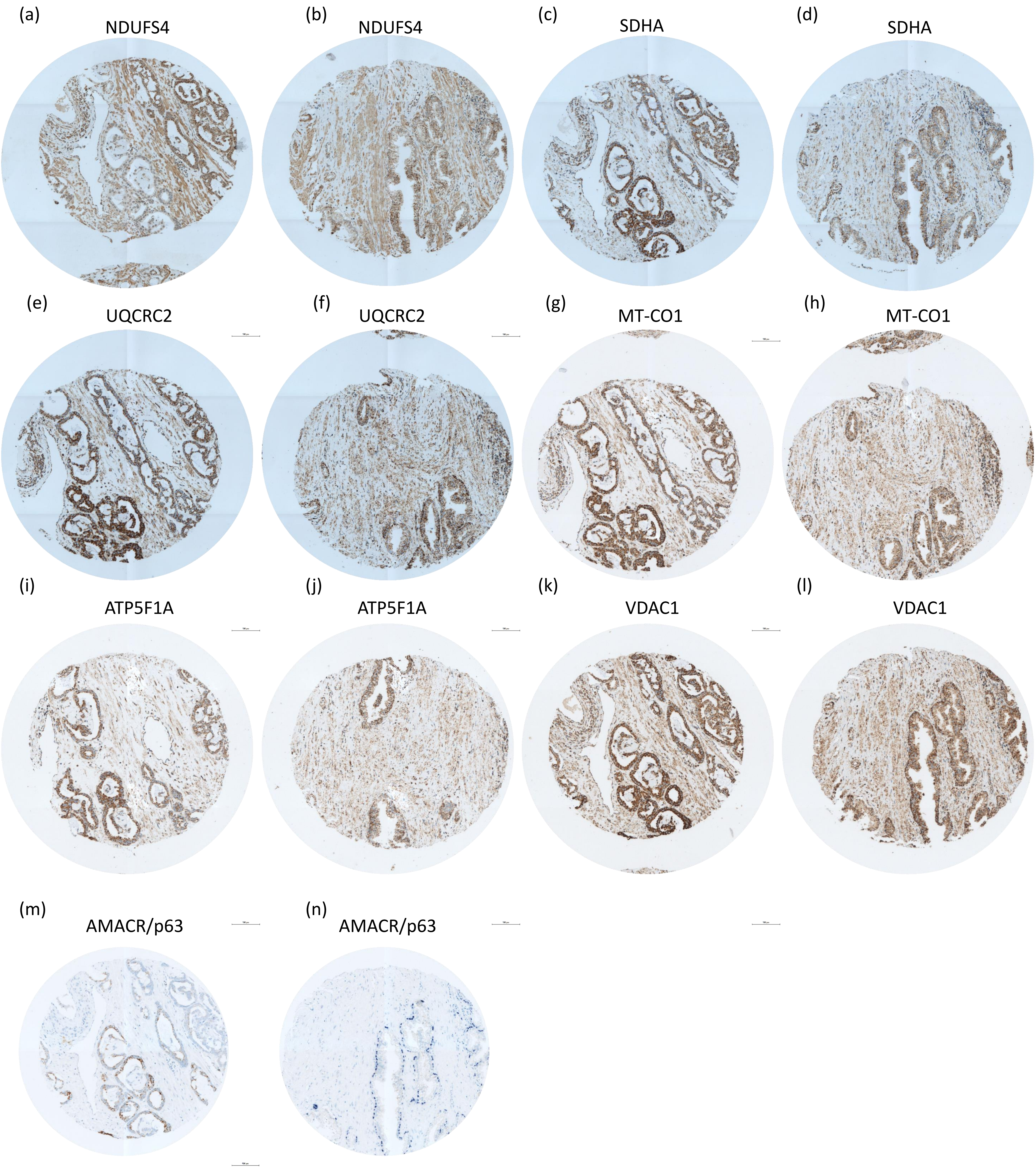

Supplementary figure 3: Staining of the OXPHOS complexes, VDAC1 and AMACR/p63 in a prostate carcinoma Gleason score 7 and the adjacent benign prostate tissue. (a, b) NDUFS4, (c, d) SDHA, (e, f) UQCRC2, (g, h) MT-CO1, (i, j) ATP5F1A, (k, l) VDAC1, (m, n) AMACR/p63. (a, c, e, g, i, k) carcinoma; (b, d, f, h, j, l, n) benign prostate tissue. The punches are 0.6 mm in diameter. The AMACR/p63 staining was used to visualize the carcinoma cells in brown and the benign prostate tissue in blue.

Supplementary table 1A: Staining intensities

		VDAC1				NDUFS4				SDHA				UQCRC2				MT-CO1				ATP5F1A			
		CA		BE		CA		BE		CA		BE		CA		BE		CA		BE		CA		BE	
Tumor	Punch	Low/High		Low/High		Low/High		Low/High		Low/High		Low/High		Low/High		Low/High		Low/High		Low/High		Low/High		Low/High	
9932197	B1	3				2	3	2	2	1	2	0	1	2	3		2	2	3						
9932197	B2			3	4			3	3			1	3				3			2	3			2	3
9935954	B3	3				2	3			1	2			1	2			2	3			2	3		
9935954	B4	3	4			2	3			1	2			1	3			2	3			2	3		
9935954	B5	2	3					1	2			1	2					1	3					2	3
9935954	B6			3				1	2			1	3			1	2	2	3			2	3		
10338726	B7	3	4			0	2			1	4			1	3			1	3						
10338726	B8			3	4			1	3			1	3			2	3			2	3			2	3
10343434	B9	3	4			2	3			2	3			1	3			2	3			2	3		
10343434	B10	3	4			2	3			2	3			3	3			2	3			2	3		
10343434	B11	3	4			2	3	2	3	3	3	2	3	3	3	2	3	2	3	2	3	2	3		
10343434	B12			3	4			1	3			1	3			2	3			1	3			1	2
10631521	B13	3	4					2	3			2	3			2	3			2	3				
10631521	B14			3	4			1	3			2	3			3	3			2	3			2	3
10637255	B15	3	4			2	3			2	2			2	3			2	3			2	3		
10637255	B16	3	4			3	3			2	2			2	3			2	3			2	3		
10637255	B17	3	4			2	3			1	2			2	3			2	3			2	3		
10637255	B18			2	3			2	3			1	2	3	3	2	3	2	3					2	3
9916972	C1	3	4			3	3			1	3			2	3			2	3						
9916972	C2	3	4			3	4			1	3			2	3			2	3			3	3		
9916972	C3	3	4			2	3			1	3			1	3			2	3			1	3		
9916972	C4			3	4			2	3			2	2			2	3							2	3
9932197	C5	3	4					1	3			2	2			2	3							2	3
9932197	C6	3	4			2	3			2	3			2	3	2	3	2	3			2	3	2	3
10325091	C7	3	4			2	3	2	3	3	3	1	3	2	3	2	3	2	3						
10325091	C8	3	4			1	3			3	3			2	3			2	3			3	3		
10325091	C9	3	4			1	3			2	3			1	3			2	3			3	3		
10325091	C10			3				2	3			2	2			3	3			2	3			2	3
10338726	C11	3																3	3	1	2			1	3
10338726	C12	3	**4**			1	3	2	3	2	3	1	2	2	3	2	3	0	1	2	3	1	3		
10630526	C13	3	4			2	3			3	4			2	3			2	3			2	3		
10630526	C14	3	4			3	3			3	3			2	3			2	3			2	3		
10630526	C15	3	4			2	4	3	3	3	4	3	3	2	3			1	3			2	3		
10630526	C16			3	4			2	3			2	3	2	3			2	3					2	3
10631521	C17	3	4			2	3			2	3			2	3			2	3			2	3		
10631521	C18	2	4			3	3	3	3	2	3	1	3		3			2	3			2	3		
10904082	C19	3	4	2		2	3	2	3	3	4	1	2	2	3			2	3			1	3		
10904082	C20	2	4			2	3			3	4			2	3			2	3			2	3		
10904082	C21	2	**4**			1	3	3	3	3	4	1	3	2	3	2	3	2	3			2	3		
10904082	C22			2	3			2	3			2	3			2	3			2	3			2	3
9908111	D1	3	4					1	2			0	1			2	3			2	3			2	3

9908111	D2			2	3			1	2			0	1			2	2			2	2			0	1
9909768	D3	3	4			1	3			1	3			3	3			3	3			2	3		
9909768	D4	4				3	4	2	2	3	4	2	3	3	3			3	3			2	3		
9909768	D5	4				1	2	3	3	3	4	1	2					2	3			2	3	2	2
9909768	D6			3	4			1	2			1	2	2	3			2	3			1	2		
10310392	D7	4				1	3			2	3			3	3			3	3						
10310392	D8			3				2	3			1	2			3	3			3	3			2	2
10311955	D9	3	4			2	3			3	3			3	3			3	3			2	3		
10311955	D10	3	4			2	3			3	3			3	3			3	3			2	3		
10311955	D11	3	4			2	3			3	3					2	3			2	3	2	3		
10311955	D12			2	3	2	2	2	3	2	3	2	3			2	3			2	3			1	3
10625132	D13	3	4			3	3	3	3	3	3	2	3	2	3			2	3			2	3	2	3
10625132	D14			3	4			2	3			1	3			2	3			2	3			2	3
10627970	D15	3				3	4			3	3			2	3			2	3			2	3		
10627970	D16	3				3	3			2	3			2	3			2	3			2	3		
10627970	D17	2	3					2	3			1	2			2	3			2	3			1	2
10627970	D18			2	4			2	3			1	3			2	3			2	3			2	2
10817500	D19	4				4	4			4	4			2	3			2	3			3	3		
10817500	D20			3	4			3	3			1	3			2	3			2	3			2	3
10821594	D21	4				2	3			1	4			1	3			1	3			3	3		
10821594	D22	3	4			2	3	2	3	2	4	0	1	1	3			1	3			1	3		
10821594	D23	2	4			2	4	2	3	2	4	0	1	1	3			1	3			2	3		
10821594	D24			3				2	3			1	2			1	3			1	3			1	3
9902802	E1	4				1	3			1	3			2	3			2	3			2	3		
9902802	E2	4				2	3	3	3	1	4	2	3	1	3			1	3					1	3
9902802	E3	2	4			2	3	2	2	1	3	1	1	2	3			2	3			2	3		
9902802	E4			2				1	2			0	1			1	2			1	2			0	1
9908111	E5	3	4			2	3			1	3							2	3			2	3		
9908111	E6	3	4			3	3	1	2	2	3	1	2	1	3	1	2	2	3			1	2		
10239624	E7	2	4			2	3			1	2			2	3			2	3						
10239624	E8	3	4			3	3			2	2			2	3			2	3			1	3		
10239624	E9	3	4			2	3			1	3			1	3			1	3			1	3		
10239624	E10			4				2	3			2	2			2	3			2	3			1	2
10310392	E11	4				2	3	1	2	3	4	1	2	2	3			2	3			2	3		
10310392	E12	3	4			3	3			3	4			2	3			2	3			2	3		
10624826	E13	4				3	3			1	2			2	3			2	3			2	3		
10624826	E14	4				4	4			1	2			2	3			2	3			2	3		
10624826	E15	2	4			3	3			1	2			2	3			2	3			2	3		
10624826	E16			3	4			1	3			1	2			2	3			2	3			1	2
10625132	E17	4				2	3			1	3			2	3			2	3			2	3		
10625132	E18	4				3	3	2	3	2	3	2	3	2	3			2	3			2	3		
10812983	E19	4				3	3			2	3			2	3			2	3			2	3		
10812983	E20	3	4			3	4			2	3			2	3			2	3			2	3		
10812983	E21	4				3	3			2	3			2	3			2	3			2	3		
10812983	E22			3				3	3			3	3			2	3			2	3			1	3
10817500	E23	3	4			2	4			3	3			2	3			2	3			2	3		

10817500	E24	3	4			3	3	3	4	3	3	3	3	3	3	3	3	0	1	2	2	3	3		
9901335	F1	2	4					1	3			0	2			1	2			1	2			1	1
9901335	F2			3	4											2	2			2	2			1	2
9901797	F3	2	4			2	3			1	3			2	3			2	3			1	3		
9901797	F4	2	4			2	3			1	3			2	3			2	3			1	3		
9901797	F5	3	4			2	3			1	3			2	3			2	3			1	3		
9901797	F6			4				2	3			2	3			2	3			2	3			3	3
10238998	F7	2	4			1	2	3	3	1	2	2	3			2	3			2	3	1	2	2	3
10238998	F8			4				2	2			2	2			2	3			2	3			2	3
10239551	F9	3	4			1	2	1	2	3	3	2	2	2	3			2	3			2	3		
10239551	F10	3	4			2	2	2	3	1	2	2	2	2	3			2	3			1	3	1	3
10239551	F11	4						1	3			2	2			2	3			2	3			2	3
10239551	F12			4																					
10613128	F13	4				3	4			3	4			2	3			2	3			2	3		
10613128	F14			4				2	3			2	2			2	3			2	3			2	3
10622130	F15	3	4			2	3	2	3	2	3	2	3	2	3	2	3	2	3	2	3	1	3	1	3
10622130	F16	4												3	3			3	3						
10622130	F17	3	4			2	3	2	3	2	3	2	3	3	3			3	3			2	3		
10622130	F18			2	4	3	3	1	2	1	1	2	3	2	3			2	3			2	3		
10736608	F19	3	4			2	3	2	2	2	3	2	2	2	3			2	3			2	3		
10736608	F20			2	4			1	2			1	2			2	3			2	3			1	3
10811752	F21	3	4			3	4			3	4			2	3			2	3			3	3		
10811752	F22	3	4			3	4			3	3			2	3			2	3			1	3		
10811752	F23	3	4			2	3			2	3			2	3			2	3			2	3		
10811752	F24			2	4			2	3			1	2			1	3			1	3			1	3
9901036	G1	4				3	3			3	4			3	3			3	3			2	3		
9901036	G2	4				1	3			1	3			1	3			1	3			0	3		
9901036	G3	4				3	4			3	4			3	3			3	3			3	3		
9901036	G4			4				2	3			2	4			2	3			2	3			2	3
9901335	G5	3	4			2	3			2	3							2	3			2	3		
9901335	G6	4										2	3	1	2	1	2	1	3			0	3	1	3
10217305	G7	1	4			1	2			1	2			1	3			1	3			0	3		
10217305	G8	2	4			2	3			2	3			1	3			1	3			0	3		
10217305	G9	2	4			1	3			1	3			1	3			1	3			0	3		
10217305	G10			3	4			2	3			2	2	2	3			2	3					2	3
10238998	G11																					2	3		
10238998	G12							1	1			1	2			1	2			1	2			2	3
10607452	G13	2	4			2	3			1	2			1	3			1	3			1	3		
10607452	G14	3	4			2	3			1	2			1	3			1	3			1	3		
10607452	G15	3	4			2	3			1	3			1	3			1	3			1	3		
10607452	G16			3	4			2	3			1	2			1	3			1	3			1	3
10613128	G17	4				4	4			3	4			2	3			2	3			3	3		
10613128	G18	4				3	4			3	4			2	3			2	3			3	3		
10734364	G19	3	4			2	3	1	2	3	4	2	3	3	3	1	2	3	3	1	2	3	3		
10734364	G20	3	4			2	3	1	1	3	4	3	3	2	3			2	3			3	3	2	3
10734364	G21	3	4			1	3	2	2	3	4	3	3	2	3			2	3			3	3		

10734364	G22			3	4			2	3			2	3	2	3			2	3					1	2
10736608	G23	3	4			2	3	2	2	3	4	2	3	2	3			2	3			2	3		
10736608	G24	3	4			1	3			2	3			1	3			1	3			1	3		
9841708	H1	3	4			2	3			2	3			1	3			1	3			3	3		
9841708	H2			3	4			1	2			1	2			1	2			1	2			1	3
9842434	H3	4				1	3			1	3			2	3			2	3			1	3		
9842434	H4	4				2	3			2	3			3	3			3	3			3	3		
9842434	H5	4				1	3			2	3			3	3			3	3			3	3		
9842434	H6			3	4			2	3			2	2			2	3			2	3			2	3
10213893	H7	3	4					2	2			2	2			2	3			2	3			2	3
10213893	H8			4		3	3	2	3	2	3	2	3			3	3			3	3			2	3
10217220	H9	3	4			1	3			1	3			1	3			1	3			0	3		
10217220	H10	3	4			1	3			1	2			1	3			1	3			1	3		
10217220	H11	3	4			3	4			3	3			3	3			3	3			2	3		
10217220	H12			3	4			2	3			1	3			1	3			1	3				
10604940	H13	4				3	4			3	4			3	3			3	3			2	3		
10604940	H14			3	4	2	3			2	2					2	3			2	3			1	3
10606082	H15	4				1	3	3	3	1	3	3	4	1	3			1	3			2	3	2	3
10606082	H16	3	4			3	3			1	2			2	3			2	3			2	3		
10606082	H17	3	4			3	3			1	2			2	3			2	3			2	3	0	1
10606082	H18			4				2	3			1	2			2	3			2	3			2	3
10729892	H19	4				2	4	3	3	2	3	1	3	2	3			2	3			1	3		
10729892	H20			3	4	2	3	3	4	2	3	1	3	2	3	2	3	2	3	2	3	2	3	0	2
10732505	H21	3	4			2	3			2	3			2	2			2	2			2	3		
10732505	H22	3	4			3	4			2	3			2	3			2	3			2	3		
10732505	H23	3	4			3	4			2	3			2	3			2	3			2	3		
10732505	H24			3	4			2	4			1	3			2	3			2	3			0	1
9839857	I1	3	4			2	3			1	3			2	3			2	3			2	3		
9839857	I2	4				1	1	2	3	2	3	2	3	3	3			3	3					2	3
9839857	I3	4						2	2			1	2			2	3			2	3			0	3
9839857	I4			4				2	3			1	2			2	3			2	3			2	3
9841708	I5					2	3			1	3			2	3			2	3			3	3		
9841708	I6	2	4			1	2	1	2	1	3	2	2	2	3			2	3			3	3		
10212172	I7																								
10212172	I8	3	4			1	2			2	3			1	3			1	3			2	3		
10212172	I9	3	4			0	2			1	2			1	3			1	3			1	3		
10212172	I10			3	4			2	3			2	3			2	3			2	3			1	3
10213893	I11	4				2	2	2	3	3	3	2	3	2	3			2	3			2	3		
10213893	I12	3	4			1	3			2	3			1	3			1	3			2	3		
10601619	I13	3	4			2	3	2	3	2	3	2	3	2	3	2	3	2	3	2	3	2	3		
10601619	I14	3	4			3	3	1	3	1	3	2	3	2	3	2	3	2	3	2	3	2	3	2	3
10601619	I15	4				3	3	2	3	2	3	2	3	2	3	2	3	2	3	2	3	2	3	1	3
10601619	I16			4				1	3			2	3			2	3			2	3			2	3
10604940	I17	4				2	4			2	4			3	3			3	3			3	3		
10604940	I18	3	4			3	3			2	4			3	3			3	3			3	3		
10729632	I19	4				3	4			2	4			3	3			3	3			3	3		

10729632	I20	4				3	4			2	4			3	3			3	3			3	3		
10729632	I21	3	4			3	4			2	3			2	3			2	3			3	3		
10729632	I22			3	4			1	1			1	2			1	3			1	3			0	1
10729892	I23	3	4			2	3			2	3			2	3			2	3			2	3	2	2
10729892	I24	3	4			2	4			2	3			2	3			2	3			2	3	2	3
9832740S	J01	3	4			1	2	1	2	3	3	1	1	3	3	1	1	3	3	1	1	3	3	1	2
9832740S	J02			3	4			1	2			1	2			1	2			1	2			1	3
9838248	J03	3	4			2	3	2	3	1	3	1	2	1	3			1	3			1	2	3	3
9838248	J04	3	4			2	3			1	2			1	3			1	3			1	2		
9838248	J05	4				2	3	1	3	1	3	1	3	2	3			2	3			1	2	2	3
9838248	J06			4				2	3			2	3			2	3			2	3			0	2
10203428	J07	4				2	3			1	4			2	3			2	3			1	3		
10203428	J08			4				2	3			1	3			2	3			2	3			1	3
10204696	J09	4						1	2			1	3			2	3			2	3			1	2
10204696	J10	3	4			2	3			2	3			2	3			2	3			2	3		
10204696	J11	4				2	3			2	4			2	3			2	3			2	3		
10204696	J12			2	4			1	2			0	1			2	3			2	3			1	2
10514489	J13	2	4			2	3			2	3	1	1	1	3			1	3			2	3	2	3
10514489	J14			2	4			1	2			1	2			1	3			1	3			1	3
10529151	J15	2	4			1	3			1	3	1	2	1	3			2	3			0	2		
10529151	J16	2	4			1	3			1	3			1	3			2	3			1	2		
10529151	J17	2	4			1	3			1	3			2	3			2	3			1	2		
10529151	J18			3	4	2	3			2	4			3	3			3	3			2	3		
10727106	J19	4				2	4			2	4			2	3			2	3			3	3		
10727106	J20			4		3	4					3	3			2	3			2	3			2	3
10728457	J21																								
10728457	J22	3	4			3	4			2	4			2	3			2	3			2	3		
10728457	J23	3	4			3	4			3	3			2	3			2	3			1	3		
10728457	J24			3	4			2	3			2	3			2	3			2	3			2	3
9832588	K1	2	4			2	3			1	3			2	3			2	3			2	3		
9832588	K2	3	4			2	3			1	3	2	3	2	3			2	3			2	3		
9832588	K3	3	4			2	3			1	2			2	3			2	3			3	3		
9832588	K4			4		2	3			2	3					1	2			1	2			0	3
9832740S	K5	4						2	3			1	3	3	3			3	3			3	3		
9832740S	K6	4				2	3			2	3			3	3			3	3			3	3		
10136806	K7	4				1	2			1	3			2	2			3	3			2	3		
10136806	K8	3	4			2	3	1	2	1	2	1	2	2	3	2	2	3	3	2	3	2	3	1	2
10136806	K9	4				2	3			1	2	1	2	3	3			3	3			2	3		
10136806	K10			4				1	2			1	2			2	3			2	3			1	2
10203428	K11	2	4			2	3			1	3			2	3			2	3			3	3		
10203428	K12	4				2	3			1	3			2	3			2	3			2	3		
10513260	K13																								
10513260	K14																								
10513260	K15	3	4			1	2			2	3			2	3			2	3			2	3	1	2
10513260	K16			2	4	2	3	0	1	3	3	1	2	2	3	1	2	2	3	1	2	2	3	1	2
10514489	K17	4				2	3			3	3			2	3			2	3			2	3	3	3

10514489	K18	4				2	3	2	3	2	3	2	3	2	3			2	3					3	3
10726906	K19	3	4					1	3			1	3			2	3			2	3			1	3
10726906	K20	4				2	3			2	3	2	3			2	3			2	3			2	3
10726906	K21	4				1	2			3	3	1	1	1	3	2	2	1	1	2	3	3	3	1	2
10726906	K22			3	4			1	2			1	2			2	3			2	3			1	2
10727106	K23	3				2	3			1	3			3	3			1	3			1	2		
10727106	K24	3	4			2	3			1	3			3	3			1	3			1	3		
9828722	L1																								
9828722	L2			2	4			0	2			1	3			2	2			2	2			1	3
9832436	L3	3	4			0	3			1	3			3	3			3	3			3	3		
9832436	L4	4				0	3			1	3			3	3			3	3			3	3		
9832436	L5	3	4			1	1			2	3			3	3			3	3			3	3		
9832436	L6			4				1	2			1	2			2	2			2	2			1	3
10115487	L7	3	4			1	3			1	2			2	3			2	3			1	3		
10115487	L8			4				1	2			1	2			2	3			2	3			2	3
10134427	L9	3	4			1	3	1	2	1	3	1	2	2	3	2	3	2	3	2	3	3	3	1	2
10134427	L10	3	4			1	3			2	3			1	3			1	3			3	3		
10134427	L11	3	4			2	3	1	3	2	3	2	3	2	3	2	3	2	3	2	3	2	3		
10134427	L12			4				1	2			1	3			2	3			2	3			1	3
10511812	L13	4				2	4			1	3			3	3			3	3			3	3		
10511812	L14			4				1	3			2	3			1	3			1	3			1	3
10512852	L15	4				2	4			3	4			2	3			2	3			2	3		
10512852	L16	2	3			2	2	1	2	2	3	2	3	2	3	2	3	2	3	2	3	2	3		
10512852	L17	2	4			2	3	2	2	2	2	2	2	1	3			1	3			1	3		
10512852	L18			3	4			2	3			1	3			1	2			1	2	1		1	3
10726179	L19	3	4			3	4			2	4			2	3	2	3	2	3	2	3	2	3		
10726179	L20			3	4											2	3	3	3						
10726359	L21	3	4			1	3	3	4	2	3	2	3	2	3			2	3			1	3	1	3
10726359	L22	3	4					2	3			2	3	1	2	2	3	1	2	2	3	1	3		
10726359	L23	3	4			3	4			2	3			2	3			2	3			2	3		
10726359	L24			3	4			3	4			2	3			2	3			2	3			2	3
9827917	M1	3	4			2	3	1	2	2	3	1	1	2	3	1	2	2	3	1	2	3	3	2	3
9827917	M2	3	4			3	4	1	3	2	3	1	1	2	3			2	3			3	3	1	3
9827917	M3	4				1	3			2	3			2	3			2	3			3	3		
9827917	M4															2	3			1	2			1	3
9828722	M5	3	4			0	1			2	3			2	2			2	2						
9828722	M6																								
10038130	M7	3	4			1	3			1	2			1	3			1	3			1	3		
10038130	M8	4						1	2			1	3	1	3			1	3					1	2
10038130	M9	2	4			1	3			1	3			1	3			1	3			1	2	2	3
10038130	M10			3	4			1	2			1	3	2	3			2	3					1	3
10115487	M11	3	4			1	1	1	1	1	1	1	1	2	3	2	3	2	3	2	3	2	3	1	3
10115487	M12	3	4			1	3			1	3			2	3			2	3			2	3		
10443208	M13							1	1			1	2			3	3			3	3			2	3
10443208	M14	3	4					1	2			1	3			3	3			3	3			2	3
10443208	M15																								

10443208	M16			3	4			1	2			1	3			2	3			2	3			1	3
10511812	M17	2	4			0	2			1	3			1	3			1	3			0	3		
10511812	M18	3	4			1	2			1	2			1	3			1	3			1	3		
10725463	M19																								
10725463	M20	4				2	3	1	1	3	4	1	2	2	3			2	3			3	3		
10725463	M21	4				3	3			3	4			2	3			2	3			3	3		
10725463	M22			3				1	2			1	2			1	3			1	3			1	3
10726179	M23	3	4			1	3			1	3			2	3			2	3			1	3		
10726179	M24	3	4			2	4			1	2			2	3			2	3			2	3		
9823371	N1	3	4											2	3			2	3			3	3	2	3
9823371	N2			3	4			2	3			2	3			2	3			2	3			2	2
9826099S	N3																								
9826099S	N4	3	4					1	2			1	1			3	3			3	3			2	2
9826099S	N5	2	4			1	2	1	2	1	2	1	2	2	2	2	3	2	2	2	3			2	2
9826099S	N6			3	4	2	3	1	2	2	3	1	2			2	3			2	3			2	3
10022186	N7	3	4			2	3			3	3			3	3			3	3			3	3		
10022186	N8															2	3							2	3
10027530	N9	3	4			1	3			2	3			2	3			2	3			1	3		
10027530	N10	1		3	4	0	3	2	3	1	2	2	2	1	3	2	3	1	3	2	3	1	3		
10027530	N11	2	4			0	3	2	3	1	2	2	2	2	3	2	3	2	3	2	3	2	3		
10027530	N12			2	3			1	2			1	2			2	2			2	2			2	3
10431981	N13	3	4			0	3			2	4	2	4	3	3			3	3			1	3		
10431981	N14			4				2	3			2	3			3	3			3	3			2	3
10433224	N15	4						1	2			2	3			2	3			2	3			1	2
10433224	N16	3	4			1	3	1	2	3	3	2	2	1	3	2	3	1	3	2	3	2	3	1	1
10433224	N17	3	4			3	4	1	1	2	3	2	2	3	3			3	3			2	3		
10433224	N18			3	4			1	1			2	3	2	3			2	3					0	3
10718773	N19	3	4			0	1			3	4			3	3			3	3						
10718773	N20			3	4			1	2			2	3			2	3			2	3			1	2
10720178	N21	3	4					2	3			3	3			2	3			2	3			2	3
10720178	N22	3	4			2	3			2	3			2	3			2	3			1	3		
10720178	N23	3	4			1	3			2	3	3	3	2	3			2	3			2	3		
10720178	N24			3	4			1	1			1	2	2	3			2	3					1	2
9823060	O1	2	4					1	3			1	2			2	3			2	3			1	3
9823060	O2	3	4			2	3	1	3	1	2	1	3	2	3	1	3	2	3	1	3	2	3	1	2
9823060	O3	4				2	3	2	3	2	3	1	2			2	3			2	3			1	2
9823060	O4			4				3	3			2	2			2	3			2	3			2	3
9823371	O5	4																							
9823371	O6	3	4			2	3	3	3	2	3	3	3	2	3	3	3	2	3	3	3			3	3
10015784	O7	4				3	3	3	3	3	3	3	3	2	3	2	3	2	3	2	3			3	3
10015784	O8	3	4			3	3			2	3			3	3			3	3			3	3		
10015784	O9	3	4			3	4			3	4			3	3			3	3			3	3		
10015784	O10			3	4			3	3			2	3			2	3			2	3			2	3
10022186	O11	3	4			3	3	3	3	2	3	2	3	3	3			3	3			3	3		
10022186	O12	3	4			3	3			3	3			3	3			3	3			3	3		
10426270	O13	3	4			2	3			1	3			1	3			1	3			1	3		

10426270	O14	2	4			1	3	1	2	1	3	1	3	1	3			1	3			1	3		
10426270	O15	2	4			1	3			1	3			1	3			1	3			1	3		
10426270	O16			3	4			3	3			2	3			3	3			3	3			1	3
10431981	O17	2	4			0	2			1	2	3	3	1	1	3	3	1	1	3	3	0	1		
10431981	O18	2	4			0	2			1	3	2	3	1	1	3	3	1	1	3	3	0	1		
10709663	O19	3	4			2	2	2	2	1	2	2	3	2	3	2	2	2	3	2	2	3	3		
10709663	O20	2	4					2	3			1	3			2	3			2	3			2	2
10709663	O21	3	4			2	3	1	2	1	3	1	3	2	3	2	3	2	3	2	3	2	3	1	2
10709663	O22			3	4			1	3			1	3			2	3			2	3			1	2
10718773	O23	3	4			1	2			2	3			3	3			3	3			2	3		
10718773	O24	3	4			1	3	1	2	2	3	1	2	3	3	2	3	3	3	2	3	2	3	1	2
9815043	P1	3	4			2	3	2	3	1	3	1	3	1	3			1	3			2	3		
9815043	P2			3	4			1	2			1	2			2	3			2	3			1	2
9818278	P3	2	3	3	4	2	3	2	3	1	3	2	2	2	3	2	3	2	3	2	3	1	3	1	2
9818278	P4	2	3	3	4	1	3	1	2	1	3	1	2	2	3	2	3	2	3	2	3	2	3	2	3
9818278	P5	3	4			2	3			1	3			3	3			3	3			2	3	1	1
9818278	P6			3	4			2	3			1	3			2	3			2	3			2	2
10003847	P7	3	4			3	4			3	3			3	3			3	3			3	3		
10003847	P8																							2	3
10006811	P9	2	4			2	3			2	3			2	3			2	3			2	2		
10006811	P10																					2	3		
10006811	P11	2	4			3	4	2	2	2	3	1	1	2	3			2	3			1	3		
10006811	P12			3	4			3	3			1	3			3	3			3	3			1	2
10400680	P13	2	4	2	4	2	3	1	2	2	3	2	2	2	3	2	3	2	3	2	3	1	3	1	3
10400680	P14			3	4			1	3			1	2			0	2			2	3	2	2		
10402495	P15	3	4			1	2			2	3			3	3			3	3			2	3		
10402495	P16	3	4			1	2	1	2	1	2	2	2	3	3			3	3			2	2		
10402495	P17	3	4			2	3			1	2			3	3			3	3			2	3		
10402495	P18			3	4			2	3			2	2			3	3			3	3				
10700693	P19	3	4			3	3			2	3			3	3			3	3			2	2		
10700693	P20			2	4	3	3	3	3	2	3	2	3	1	3	2	3	1	3	2	3	1	3	1	3
10701686	P21	2	4			3	3			2	3			2	3			2	3			2	2		
10701686	P22	3	4			3	4	3	3	3	4	3	3	2	3			2	3			2	2		
10701686	P23	3	4			3	3	3	4	2	4	3	4	2	3			2	3			2	3		
10701686	P24			3	4			2	3			1	3			2	3			2	3			2	2
9740516	Q1	1	4			1	3			0	2			0	3			0	3			0	2		
9740516	Q2	3	4			1	3			1	3			2	3			2	3			1	3		
9740516	Q3	3	4			2	3			1	3			1	3			1	3			1	3		
9740516	Q4			2	4	1	3			1	2			1	3			1	3			1	2		
9815043	Q5	2	4			2	2	2	3	2	3	2	3	3	3	3	3	3	3	3	3	1	3		
9815043	Q6	2	4			2	3			1	3			3	3			3	3			1	3		
9940301	Q7	4				1	2			3	4			1	2			3	3			3	3		
9940301	Q8	4				1	2			3	4			3	3			3	3			3	3		
9940301	Q9	4				1	2			3	4			1	2			3	3			3	3		
9940301	Q10			4				1	3			2	3			3	3			3	3			2	3
10003847	Q11	4				2	3	1	2	3	3	1	2	3	3	3	3	3	3	3	3	2	3	1	3

10003847	Q12																					3	3	1	1
10345627	Q13	4				2	2			2	3			3	3			3	3			2	2		
10345627	Q14	3	4			2	3			3	3					2	3			2	3	2	3		
10345627	Q15	3	4			2	3			3	3			3	3			3	3			2	3		
10345627	Q16			3	4			2	3			1	3			2	3			2	3			2	2
10400680	Q17	3	4					2	3			1	3			2	3			2	3			1	3
10400680	Q18	2	4			1	2	2	3	0	3	1	3	0	1	2	3	0	1	2	3			2	3
10639881	Q19	2	3			1	3			1	3	2	2	1	3	2	3	1	3	2	3	1	3	1	3
10639881	Q20	3	4			1	3	1	3	3	4	2	3	2	3	2	3	2	3	2	3	1	3	1	3
10639881	Q21	3	4			1	3			1	3			3	3			3	3			3	3	1	2
10639881	Q22			2	3	1	1	2				2	2			1	3			1	3			1	2
10700693	Q23	3	4			3				3	3			3	3			3	3			1	2		
10700693	Q24	3	4			3				2	3			3	3			3	3			1	3		

CA: Prostate carcinoma; BE: benign tissue. The intensities are given as 0=no staining, 1=weak, 2=moderate, 3=strong, 4=very strong. Low=lowest staining intensity of a tissue punch. High= highest staining inensity of a tissue punch.

Supplementary table 1B: Mean staining intensities and clinical data

Tumor	VDAC1		NDUFS4		SDHA		UQCRC2		MT-CO1		ATP5F1A		Age at diagnosis [years]	Age at radical prostatectomy [years]	Gleason score	Gleason pattern 1 at RPE	Gleason pattern 2 at RPE	Resection border; 0=witout tumor, 1=tumor positive	Tumor stage at RPE	Prostate volume at RPE	PSA levels at diagnosis	fPSA% at diagnosis	PSA progression	Time to PSA progression
	CA	BE	CA	BE	CA	BE	CA	BE	CA	BE	CA	BE												
9740516	3,2	3,0	2,1		1,6		2,0		2,0		1,8		67	67	5	2	3	0	2b	47	8,5	14,6	unknown	0
9815043	3,2	3,5	2,3	2,2	2,2	2,0	2,7	2,8	2,7	2,8	2,2	1,5	68	69	6	3	3	0	2b	26	7,3	12,6	unknown	0
9818278	2,8	3,5	2,3	2,2	2,0	1,8	2,7	2,5	2,7	2,5	2,3	1,8	53	53	7	3	4	0	2a	24,5	2,2	10,5	no	0
9823060	3,5		2,5	2,4	2,0	1,8	2,5	2,4	2,5	2,4	2,5	1,9	57	57	5	2	3	0	2c	20	2,6	12,3	no	0
9823371	3,7	3,5	2,5	2,8	2,5	2,8	2,5	2,8	2,5	2,8	3,0	2,5	55	55	8	4	4	0	2b	25	7,4	7,6	unknown	0
9826099S	3,3	3,5	2,0	1,5	2,0	1,3	2,0	2,7	2,0	2,7		2,2	57	58	5	3	2	0	2c	25	7,9	8,7	unknown	0
9827917	3,7		2,7	1,8	2,5	1,0	2,5	2,0	2,5	1,5	3,0	2,2	69	69	6	3	3	0	3a	26	6,8	16,3	unknown	0
9828722	3,5	3,0	0,5	1,0	2,5	2,0	2,0	2,0	2,0	2,0		2,0	56	56	6	3	3	0	3a	17	3,1	4,5	noRad	0
9832436	3,7		1,3	1,5	2,2	1,5	3,0	2,0	3,0	2,0	3,0	2,0	52	52	6	3	3	0	2b	20	2,9	15,2	unknown	0
9832588	3,3		2,5		2,0	2,5	2,5	1,5	2,5	1,5	2,7	1,5	57	58	7	4	3	0	2c	50	12,4	14,9	no	0
9832740S	3,8	3,5	2,0	1,8	2,8	1,5	3,0	1,3	3,0	1,3	3,0	1,8	59	59	7	3	4	0	3a	20	2,1	16	noRad	0
9838248	3,7		2,5	2,3	1,8	2,0	2,2	2,5	2,2	2,5	1,5	2,2	54	54	6	3	3	0	2c	17	7,8	10	no	0
9839857	3,8		1,8	2,3	2,3	1,8	2,8	2,5	2,8	2,5	2,5	2,2	67	68	6	3	3	0	2c	17	4,1	13,7	no	0
9841708	3,3	3,5	2,2	1,5	2,2	1,8	2,3	1,5	2,3	1,5	3,0	2,0	72	73	7	3	4	0	3a	22	4,6	16,5	unknown	0
9842434		3,5	2,2	2,5	2,3	2,0	3,0	2,5	3,0	2,5	3,0	2,5	57	57	5	2	3	0	2b	40	6,2	13,2	no	0
9901036			2,8	2,5	3,0	3,0	3,0	2,5	3,0	2,5	2,8	2,5	52	52	6	3	3	0	2c	28	3,6	13,9	yes	3676
9901335	3,5	3,5	2,5	2,0	2,5	1,8	1,5	1,7	2,3	1,8	2,0	1,5	57	57	5	2	3	0	2b	31	8,2	11,2	no	0
9901797	3,2		2,5	2,5	2,0	2,5	2,5	2,5	2,5	2,5	2,0	3,0	55	55	6	3	3	1	2c	28,5	8,3	6,3	no	0
9902802	3,7		2,3	2,2	2,2	1,3	2,3	1,5	2,3	1,5	2,5	1,3	50	50	7	3	4	0	2c	18	8,5	3,4	unknown	0
9908111	3,5	2,5	2,8	1,5	2,3	0,8	2,0	2,0	2,5	2,3	2,0	1,5	54	54	5	3	2	0	2c	30	4,2	21,7	unknown	0
9909768	3,8	3,5	2,3	2,2	3,0	1,8	2,8		2,8		2,3	2,0	48	48	6	3	3	0	2a	22	2	9,5	no	0
9916972	3,5	3,5	3,0	2,5	2,0	2,0	2,3	2,5	2,5		2,5	2,5	49	50	5	2	3	0	2b	40	4,5	13,1	no	0
9932197	3,5	3,5	2,5	2,3	2,0	1,5	2,5	2,4	2,5	2,5	2,5	2,5	60	61	5	3	2	0	2c	n.a.	5,8	15	no	0
9935954	3,1		2,5	1,5	1,5	1,8	1,8	1,5	2,4		2,5	2,5	64	65	6	3	3	0	2a	42	2,1	17,6	unknown	0
9940301			1,5	2,0	3,5	2,5	2,0	3,0	3,0	3,0	3,0	2,5	68	68	8	3	5	0	2b	20	44,55	n.a.	yes	351
10003847	3,8		3,0	1,5	3,0	1,5	3,0	3,0	3,0	3,0	2,8	1,8	57	57	7	3	4	0	2c	24,8	8,2	12	no	0
10006811	3,0	3,5	3,0	2,5	2,5	1,5	2,5	3,0	2,5	3,0	2,2	1,5	49	49	6	3	3	0	2c	16,9	4,3	7	no	0
10015784	3,7	3,5	3,2	3,0	3,0	2,8	2,8	2,5	2,8	2,5	3,0	2,8	68	68	7	3	4	1	3a	30	5,8	11,9	no	0
10022186	3,5		2,8	3,0	2,8	2,5	3,0	2,5	3,0		3,0	2,5	52	52	7	3	4	0	2a	18	6	9	no	0
10027530	3,0	3,0	1,7	2,2	1,8	1,8	2,5	2,3	2,5	2,3	2,3	2,5	59	59	7	3	4	x	2c	27	9	9,7	no	0
10038130	3,5	3,5	2,0	1,5	1,8	2,0	2,2		2,2		1,8	2,0	62	62	5	2	3	0	2c	n.a.	5,4	12	no	0
10115487	3,5		1,7	1,3	1,5	1,3	2,5	2,5	2,5	2,5	2,3	2,3	56	56	5	2	3	0	3a	30	5,03	11,4	unknown	0
10134427	3,5		2,2	1,7	2,3	2,0	2,5	2,5	2,5	2,5	2,8	1,8	62	62	7	3	4	0	3a	n.a.	5,4	0	no	0
10136806	3,8		2,2	1,5	1,7	1,5	2,5	2,3	3,0	2,5	2,5	1,5	55	56	6	3	3	0	2a	25	2,8	15	no	0
10203428	3,7		2,5	2,5	2,2	2,0	2,5	2,5	2,5	2,5	2,5	2,0	58	58	7	4	3	0	2c	21	14,6	6,8	no	0
10204696	3,8	3,0	2,5	1,5	2,8	1,3	2,5	2,5	2,5	2,5	2,5	1,5	60	60	7	4	3	0	2c	29	2,5	14	no	0
10212172	3,5	3,5	1,3	2,5	2,0	2,5	2,0	2,5	2,0	2,5	2,0	2,0	65	65	6	3	3	0	2b	36,6	5,5	23,8	unknown	0
10213893	3,7		2,3	2,3	2,7	2,3	2,5	2,8	2,5	2,8	2,5	2,5	55	55	5	3	2	0	2c	46	3,3	12	no	0
10217220	3,5	3,5	2,5	2,5	2,2	2,0	3,0	2,0	3,0	2,0	2,5		72	72	7	3	4	1	2b	21	7,79	16,8	yes	2093
10217305	2,8	3,5	2,0	2,5	2,0	2,0	2,5		2,5			2,5	63	63	6	3	3	0	2b	28	6,28	6,1	no	0
10238998	3,0		1,5	2,0	1,5	2,0		2,2		2,2	2,0	2,5	51	52	6	3	3	0	2c	36	2,04	13,2	no	0
10239551	3,7		1,8	2,0	2,3	2,0	2,5	2,5	2,5	2,5	2,3	2,3	57	57	8	3	5	0	2a	35	3,68	12	no	0
10239624	3,3		2,7	2,5	1,8	2,0	2,3	2,5	2,3	2,5	2,0	1,5	67	68	5	2	3	0	2c	75	16,45	23,7	no	0
10310392	3,8		2,5	2,0	3,2	1,5	2,7	3,0	2,7	3,0	2,5	2,0	56	57	7	3	4	1	2c	30	1,85	15,1	no	0
10311955	3,5	2,5	2,4	2,5	2,9	2,5	3,0	2,5	3,0	2,5	2,5	2,0	61	61	7	3	4	1	2b	26	5,16	8,7	no	0
10325091	3,5		2,2	2,5	2,8	2,0	2,3	2,8	2,5	2,5	3,0	2,5	58	59	8	3	5	0	3a	24	2,31	15,6	no	0
10338726	3,5	3,5	1,5	2,3	2,5	1,8	2,3	2,5	1,9	2,2	2,0	2,3	56	56	6	3	3	0	2c	23	3,19	12,5	no	0
10343434	3,5	3,5	2,5	2,3	2,7	2,3	2,7	2,5	2,5	2,3	2,5	1,5	55	55	7	3	4	0	2c	35	3,15	12,1	unknown	0
10345627	3,7	3,5	2,3	2,5	2,8	2,0	3,0	2,5	3,0	2,5	2,3	2,0	52	52	5	3	2	0	2a	33	4,9	13	no	0
10400680	3,2	3,3	2,0	2,1	2,0	1,9	1,5	2,0	1,5	2,5	2,0	2,2	61	61	5	3	2	0	2c	80	9,24	17,2	no	0
10402495	3,5	3,5	1,8	2,0	1,8	2,0	3,0	3,0	3,0	3,0	2,3		60	61	6	3	3	1	2c	50	10,78	7,1	no	0
10426270	3,2	3,5	2,2	2,3	2,0	2,3		3,0		3,0		2,0	64	65	8	3	5	0	2c	30	7,61	11	no	0
10431981	3,2		1,2	2,5	2,2	2,8	1,7	3,0	1,7	3,0	1,0	2,5	58	58	8	3	5	0	2c	24	4,63	6,5	no	0
10433224	3,7	3,5	2,8	1,3	2,8	2,3	2,8	2,5	2,8	2,5	2,5	1,3	59	59	6	3	3	0	2a	28	4,21	12,1	no	0
10443208	3,5	3,5		1,3		1,8		2,8		2,8		2,3	58	59	6	3	3	0	2c	60	6,44	11,3	unknown	0

10511812	3,5		1,8	2,0	1,8	2,5	3,0	2,0	3,0	2,0	3,0	2,0	70	71	9	5	4	0	3a	30	8,23	17,7	yes	435
10512852	3,1	3,5	2,5	2,0	2,7	2,2	2,3	2,0	2,3	2,0	2,3	2,0	56	56	7	4	3	1	2c	30	6,66	15,5	yes	1319
10513260	3,5	3,0	2,0	0,5	2,8	1,5	2,5	1,5	2,5	1,5	2,5	1,5	65	66	8	5	3	0	2c	20	4,63	8,2	no	0
10514489	3,7	3,0	2,5	2,0	2,7	1,7	2,3	2,0	2,3	2,0	2,5	2,6	61	61	8	3	5	0	2c	34	2,52	10,6	no	0
10529151	3,0	3,5	2,1		2,3	1,5	2,4		2,6		1,6		56	56	7	3	4	0	2b	33	29,2	8	unknown	0
10601619	3,7		2,8	2,3	2,3	2,5	2,5	2,5	2,5	2,5	2,5	2,3	67	67	7	3	4	1	2c	30	7,11	10,1	no	0
10604940	3,8	3,5	3,0		2,9		3,0	2,5	3,0	2,5	2,8	2,0	71	71	7	3	4	0	2c	62	6,19	15,5	no	0
10606082	3,7		2,7	2,8	1,7	2,5	2,5	2,5	2,5	2,5	2,5	1,8	57	57	8	3	5	1	2c	30	6,28	12	no	0
10607452	3,3	3,5	2,5	2,5	1,7	1,5		2,0		2,0		2,0	64	64	6	3	3	0	2c	35	4,27	15	no	0
10613128			3,7	2,5	3,5	2,0	2,5	2,5	2,5	2,5	2,8	2,5	62	62	9	4	5	1	2c	42	3,46	28,3	yes	1087
10622130	3,7	3,0	2,7	2,2	2,0	2,5	2,8	2,5	2,8	2,5	2,3	2,0	59	60	5	3	2	0	2c	28,5	4,84	13	no	0
10624826	3,7	3,5	3,3	2,0	1,5	1,5	2,5	2,5	2,5	2,5	2,5	1,5	67	67	9	4	5	0	2c	30	9,1	8	yes	894
10625132	3,8	3,5	2,8	2,7	2,5	2,3	2,5	2,5	2,5	2,5	2,5	2,5	67	67	5	3	2	0	2c	40	9,03	14,5	no	0
10627970	2,8	3,0	3,3	2,5	2,8	1,8	2,5	2,5	2,5	2,5	2,5	1,8	70	70	7	3	4	0	2c	30	5,8	5,8	unknown	0
10630526	3,5	3,5	2,8	2,8	3,3	2,8	2,5		2,5		2,5	2,5	66	66	7	3	4	0	3b	40	16,9	0	unknown	0
10631521	3,3	3,5	2,8	2,5	2,5	2,3	2,5	2,8	2,5	2,5	2,5	2,5	60	61	6	3	3	0	2c	30	3,5	15,3	no	0
10637255	3,5	2,5	2,7	2,5	1,8	1,5	2,6	2,5	2,5		2,5	2,5	63	63	7	3	4	1	2c	42	3,77	17	yes	970
10639881	3,2	2,5	1,8	2,3	2,5	2,2	2,5	2,3	2,5	2,3	2,3	1,8	64	64	6	3	3	0	2c	41	3,64	14	unknown	0
10700693	3,5	3,0	3,0	3,0	2,6	2,5	2,8	2,5	2,8	2,5	1,9	2,0	57	57	5	3	2	0	2c	30	4,88	11,6	no	0
10701686	3,3	3,5	3,2	3,0	3,0	2,8	2,5	2,5	2,5	2,5	2,2	2,0	67	67	9	4	5	0	3a	20	6,91	6,1	no	0
10709663	3,3	3,5	2,3	2,0	1,8	2,1	2,5	2,4	2,5	2,4	2,8	1,7	65	65	9	4	5	0	3a	38,9	2,65	17,1	no	0
10718773	3,5	3,5	1,3	1,5	2,8	2,0	3,0	2,5	3,0	2,5	2,5	1,5	66	67	7	4	3	0	3a	25	6,1	20,1	yes	1261
10720178	3,5	3,5	2,3	1,8	2,5	2,5	2,5	2,5	2,5	2,5	2,3	2,0	59	60	7	3	4	0	2c	28	3,21	8,1	no	0
10725463			2,8	1,3	3,5	1,5	2,5	2,0	2,5	2,0	3,0	2,0	70	70	5	3	2	0	2a	30	3,18	11,2	no	0
10726179	3,5	3,5	2,8		2,2		2,5	2,5	2,6	2,5	2,3		51	52	9	5	4	1	3b	30	15,2	12	yes	153
10726359	3,5	3,5	2,8	3,2	2,5	2,5	2,2	2,5	2,2	2,5	2,2	2,3	57	57	7	3	4	1	2c	47	9,85	7,6	yes	513
10726906	3,8	3,5	2,0	1,8	2,8	1,8	2,0	2,4	1,0	2,5	3,0	1,9	67	68	5	3	2	0	2c	63	7,27	39,9	no	0
10727106	3,7		2,9		2,3	3,0	2,8	2,5	2,2	2,5	2,2	2,5	65	66	9	5	4	1	3b	80	8,6	0	yes	63
10728457	3,5	3,5	3,5	2,5	3,0	2,5	2,5	2,5	2,5	2,5	2,3	2,5	67	68	7	3	4	0	3a	30	5,52	13	no	0
10729632	3,8	3,5	3,5	1,0	2,8	1,5	2,8	2,0	2,8	2,0	3,0	0,5	64	65	7	3	4	1	2c	30	5,63	15,1	no	0
10729892	3,7	3,5	2,8	3,3	2,5	2,0	2,5	2,5	2,5	2,5	2,4	1,8	69	69	7	4	3	1	3a	30	3,39	12	yes	414
10732505	3,5	3,5	3,2	3,0	2,5	2,0	2,3	2,5	2,3	2,5	2,5	0,5	62	63	7	3	4	1	2c	n.a.	7,24	0	unknown	0
10734364	3,5	3,5	2,3	1,8	3,5	2,8	2,6	1,5	2,6	1,5	3,0	2,0	60	60	7	3	4	1	3a	25	6,45	9,9	unknown	0
10736608	3,5	3,0	2,3	1,8	2,8	2,0	2,3	2,5	2,3	2,5	2,3	2,0	65	65	7	3	4	0	3a	35	7,9	0	unknown	0
10811752	3,5	3,0	3,2	2,5	3,0	1,5	2,5	2,0	2,5	2,0	2,5	2,0	67	68	8	4	4	1	3b	50	20,75	10,46	noMed	0
10812983	3,8		3,2	3,0	2,5	3,0	2,5	2,5	2,5	2,5	2,5	2,0	67	67	9	5	4	0	3b	28	5,9	15	no	0
10817500	3,7	3,5	3,3	3,3	3,3	2,5	2,5	2,8	2,2	2,3	2,8	2,5	72	72	9	4	5	1	3b	40	18,09	8,96	unknown	0
10821594	3,5		2,7	2,5	2,8	0,8	2,0	2,0	2,0	2,0	2,5	2,0	50	51	9	5	4	0	2c	25	6,9	5,22	unknown	0
10904082	3,2	2,5	2,3	2,7	3,5	2,0	2,5	2,5	2,5	2,5	2,5	2,5	59	59	8	3	5	0	2c	50	4,4	10	unknown	0

Mean staining intensities of tumors (CA) and benign tissue (BE). n.a. = not available; noRAD = no PSA progression, patient received immediately after the radical prostatectomy a radiation therapy. It was indicated when the tumor had a high Gleason score and high risk of progression. noMED = no PSA Progression, same as noRAD with drug therapy (often short androgen withdrawal); RPE = radical prostatecomy; PSA = prostate specific antigen; fPSA% = percent free PSA compared to total PSA.

Supplementary table 2: Comparison of the expression of the OXPHOS subunits and VDAC1 in benign prostate hyperplasia and carcinomas in different Gleason scores

	Hyperplasia			Carcinoma			
	mean	SEM	n	mean	SEM	p value	n
**VDAC1**							
Gleason score 5	3.307	0.084	14	3.512	0.045	0.0316	17
Gleason score 6	3.385	0.083	13	3.419	0.065	0.7467	21
Gleason score 7	3.333	0.065	24	3.488	0.050	0.0605	32
Gleason score 8	3.083	0.154	6	3.490	0.069	0.0153	10
Gleason score 9	3.500	0.000	5	3.556	0.060	0.5132	9
**NDUFS4**							
Gleason score 5	2.106	0.121	18	2.416	0.089	0.044	19
Gleason score 6	2.045	0.103	22	2.114	0.140	0.6921	21
Gleason score 7	2.221	0.108	29	2.572	0.086	0.0126	32
Gleason score 8	2.236	0.197	11	2.191	0.169	0.8624	11
Gleason score 9	2.538	0.183	8	2.920	0.175	0.1535	10
**SDHA**							
Gleason score 5	1.850	0.104	18	2.247	0.113	0.0145	19
Gleason score 6	1.945	0.091	22	2.214	0.099	0.0522	21
Gleason score 7	2.039	0.083	31	2.597	0.075	<0.0001	32
Gleason score 8	2.145	0.142	11	2.636	0.173	0.0403	11
Gleason score 9	2.244	0.244	9	2.470	0.213	0.4940	10
**UQCRC2**							
Gleason score 5	2.376	0.069	17	2.337	0.099	0.7498	19
Gleason score 6	2.410	0.086	20	2.521	0.080	0.3494	19
Gleason score 7	2.303	0.080	30	2.609	0.045	0.0012	32
Gleason score 8	2.509	0.149	11	2.330	0.087	0.3237	10
Gleason score 9	2.420	0.077	10	2.530	0.080	0.3365	10
**MT-CO1**							
Gleason score 5	2.438	0.060	16	2.363	0.111	0.5811	19
Gleason score 6	2.411	0.085	19	2.558	0.080	0.2170	19
Gleason score 7	2.282	0.085	28	2.606	0.044	0.0009	32
Gleason score 8	2.482	0.146	11	2.450	0.100	0.8619	10
Gleason score 9	2.370	0.065	10	2.450	0.086	0.4676	10
**ATP5F1A**							
Gleason score 5	2.083	0.082	18	2.328	0.094	0.0578	18
Gleason score 6	2.110	0.092	21	2.339	0.089	0.0844	18
Gleason score 7	1.900	0.096	30	2.550	0.054	<0.0001	32
Gleason score 8	2.245	0.110	11	2.480	0.184	0.2766	10
Gleason score 9	2.078	0.120	9	2.560	0.088	0.0044	10

The mean ± SEM of the staining intensities, the p values and the number of the analyzed samples (n) is given.
